# Neonatal outcomes among twins following assisted reproductive technology: an Australian population-based retrospective cohort study

**DOI:** 10.1186/s12884-018-1949-0

**Published:** 2018-08-08

**Authors:** Alex Y. Wang, Nadom Safi, Fathalla Ali, Kei Lui, Zhuoyang Li, Mark P. Umstad, Elizabeth A. Sullivan

**Affiliations:** 1 Faculty of Health, Australian Centre of Public and Population Health Research, University of Technology Sydney, PO Box 123 Broadway, Ultimo, NSW 2007 Australia; 2 School of Women’s and Children’s Health, the University of New South Wales, Sydney, NSW 2052 Australia; 3 Department of Obstetrics and Gynaecology, Royal Women’s Hospital, University of Melbourne, Parkville, VIC 3052 Australia

**Keywords:** Assisted reproductive technology, Twins, Neonatal death, Premature birth, Stillbirth

## Abstract

**Background:**

While their incidence is on the rise, twin pregnancies are associated with risks to the mothers and their babies. This study aims to investigate the likelihood of adverse neonatal outcomes of twins following assisted reproductive technology (ART) compared to non-ART twins.

**Methods:**

A retrospective population study using the Australian National Perinatal Data Collections (NPDC) which included 19,662 twins of ≥20 weeks gestational age or ≥ 400 g birthweight in Australia. Maternal outcomes and neonatal outcomes (preterm birth, low birth weight, resuscitation and neonatal death) were compared. Generalized Estimating Equations were used to assess the likelihood of any neonatal outcomes, with adjusted odds ratio (AOR) and 95% confidence intervals (CI) presented. Weinberg’s differential rule was used to estimate monozygotic twin rate.

**Results:**

ART mothers were 3.3 years older than non-ART mothers. The rates of pregnancy-induced hypertension and gestational diabetes were significantly higher for ART mothers than non-ART mothers (12.2% vs. 8.4%, *p* <  0.01) and (9.7% vs. 7.5%, *p* <  0.01) respectively. The incidence of monozygotic twins was 2.0% for ART twins and 1.1% for non-ART twins. Compared with non-ART twins, ART twins had higher rates of preterm birth (AOR 1.13, 95% CI: 1.05–1.22), low birth weight (AOR 1.13, 95% CI: 1.05–1.22), and resuscitation (AOR 1.26, 95% CI: 1.17–1.36). Liveborn ART twins had 28% (AOR 1.28, 95% CI 1.09–1.50) increased odds of having any adverse neonatal outcome compared to liveborn non-ART twins, especially for opposite-sex ART twins (AOR 1.42, 95% CI 1.11–1.82).

**Conclusion:**

As ART twins had higher rates of adverse outcome, special prenatal care is recommended. Couples accessing ART should be fully informed of the risk of adverse outcome of twin pregnancies.

## Background

The rate of twin birth has been increased in the past four decades. In the United States, the twin birth rate has increased by 76%; from 1.9% in 1980 to 3.3% in 2009 [1]. In Australia, the rate of multiple deliveries has risen by 15% from 1.3% in 1992 to 1.5% in 2012 [2, 3]. One of the main reasons behind twining rate increase is the introduction of assisted reproductive technology (ART) [4]. The rate of twin birth is high among ART conceived children representing 43% in the US [5] and 6.8% in Australia and New Zealand [6].

Irrespective of the method of conception, twin pregnancies have a greater risk of maternal and neonatal complications compared with singleton pregnancies [7]. When the literature is limited to twins, it is inconsistent with some studies reporting comparable maternal and neonatal outcomes between ART and non-ART twin births [8] while the others have found higher rates of maternal and neonatal complications among ART twin births [4, 9]. There is a lack of national population-based studies in Australia comparing ART and non-ART twins. The aim of this study is to investigate the association of ART on the neonatal outcomes among twins in Australia.

## Methods

A national population-based retrospective cohort design was used. The data source of this study is the Australian National Perinatal Data Collection (NPDC) from 2007 to 2011. The NPDC includes information on mothers and babies of all live births and stillbirths of at least 20 weeks gestation or at least 400 g birthweight [10]. Data on whether the pregnancy resulted from ART were available for five out of the eight states and territories in Australia (Victoria, Queensland, Western Australia, Tasmania and the Australian Capital Territory) and were included in this study. These five states and territories represented approximately 60% of all births in Australia, with data validation conducted at both state and national levels [10]. The study population included 9831 sets of twin deliveries, with 4580 (23.3%) ART twins and 15,082 (76.7%) non-ART twins.

Maternal characteristics included maternal age at delivery, pre-pregnancy body mass index (BMI), parity, smoking during pregnancy, socioeconomic status, the Indigenous status of the mother, and admitted patient elected accommodation status (public/private). Pre-existing maternal conditions included pre-existing hypertension, and pre-existing diabetes. Pregnancy complications included antepartum haemorrhage, pregnancy-induced hypertension, and gestational diabetes. In NPDU, pregnancy-induced hypertension included both gestational hypertension, and pre-eclampsia [10]. Gestational hypertension is the blood pressure exceeding 140/90 mmHg during pregnancy (after 20 weeks’ gestation) on two readings at least, of more than six hours apart without pre-existing hypertension [11]. Preeclampsia is defined as the presence of hypertension accompanied by proteinuria, utero-placental or organ dysfunctions [11]. Gestational diabetes is the condition where diabetes is first diagnosed during pregnancy and may disappear after giving birth but considered as a risk factor for diabetes occurring in the future [10].

Outcomes at birth included delivery method, preterm birth (gestational age < 37 weeks), stillbirth, low birthweight (birthweight < 2500 g), Apgar score at 5 min, resuscitation at birth, admission to neonatal intensive care unit or special care nursery (NICU/SCN), length of infant stay in hospital in completed day and neonatal death (within 28 days of birth). Among liveborn twins, a combined measure ‘any adverse neonatal outcome’ was created if a birth was preterm, low birthweight, Apgar score at 5 min < 7, required resuscitation, admitted to NICU/SCN or neonatal death. Neonatal death is defined as the death of a live born baby within 28 days of birth [10].

Maternal characteristics, pre-existing maternal conditions, pregnancy complications and neonatal outcomes were compared between ART and non-ART twins. Chi-squared test for categorical variables and Student’s t-test for continuous variables were used for the comparison. Generalized Estimating Equations was used to assess the likelihood of ‘any adverse neonatal outcome’ following ART treatment, with odds ratio, adjusted odds ratio (AOR) (adjusted for maternal age, parity, Indigenous status of mother, pre-pregnancy BMI, and smoking during pregnancy) and 95% confidence intervals (CI) presented. Due to high proportion of missing data BMI and smoking during pregnancy, a sensitivity analysis was conducted by excluding smoking or BMI in the adjustment. Weinberg’s differential rule was used to estimate monozygotic twin rate [12]. Data were analysed using Statistical Package for Social Sciences (SPSS) software, version 22 (SPSS, Inc., Chicago, IL, USA).

## Results

Table 1 shows maternal demographic characteristics pre-existing conditions and pregnancy complications of ART and non-ART groups. ART mothers were 3.3 years older than non-ART mothers. Compared to non-ART mothers, higher proportions of ART mothers were primiparous, non-smoking, with normal BMI and with private health insurance. One in four ART mothers was from the least 20% disadvantaged group compared to < 20% of non-ART mothers (Table 1).

**Table 1:** Demographics, pre-existing conditions and pregnancy complications of mothers of ART and non-ART twin sets, Australia, 2007–2011

	Non-ART mothers (*n* = 7541)		ART mothers (*n* = 2290)		*P* value (chi-squared test)
	No.	%	No.	%	
Maternal age, years					
Mean ± SD	30.6 ± 5.4		33.9 ± 4.6		< 0.01*
< 30	3151	41.8	380	16.6	< 0.01
30–34	2451	32.5	874	38.2	
35–39	1678	22.3	795	34.7	
40–44	253	3.4	184	8.0	
≥ 45	8	0.1	57	2.5	
Pre-pregnancy BMI, kg/m^2^					
< 20	124	1.6	36	1.6	0.44
20–24.9	516	6.8	213	9.3	
25–29.9	424	5.6	157	6.9	
30 +	334	4.5	115	5.0	
Not stated	6134	81.3	1769	77.2	
Parity					
Primiparous	2773	36.8	1460	63.8	< 0.01
Multiparous	4768	63.2	830	36.2	
Smoking during pregnancy					
Smoked	1023	13.6	47	2.1	< 0.01
Did not smoke	4533	60.1	1709	74.6	
Not stated	1985	26.3	534	23.3	
Indigenous status of mothers					
Non-Indigenous	7239	96.0	2280	99.6	< 0.01
Indigenous	295	3.9	9	0.4	
Not stated	7	0.1	1	0.0	
Admitted patient elected accommodation status					
Public	5014	66.5	612	26.7	< 0.01
Private	2519	33.4	1676	73.2	
Not stated	8	0.1	2	0.1	
Pre-existing hypertension					
Yes	82	1.1	34	1.5	0.13
No	7394	98.1	2242	97.9	
Not stated	65	0.9	14	0.6	
Pre-existing diabetes					
Yes	42	0.6	13	0.6	0.96
No	7431	98.5	2262	98.8	
Not stated	68	0.9	15	0.7	
Antepartum haemorrhage					
Yes	340	4.5	130	5.7	0.02
No	7201	95.5	2160	94.3	
Pregnancy-induced hypertension					
Yes	634	8.4	280	12.2	< 0.01
No	6907	91.6	2010	87.8	
Gestational diabetes					
Yes	567	7.5	222	9.7	< 0.01
No	6974	92.5	2068	90.3	

^*^independent samples t-test

Antepartum hemorrhage rate was higher for ART mothers than for non-ART mothers (5.7% vs. 4.5%, *p* = 0.02). The rates of pregnancy-induced hypertension and gestational diabetes were also higher for ART mothers than for non-ART mothers (12.2% vs. 8.4%, *p* <  0.01) and (9.7% vs. 7.5%, p <  0.01) respectively (Table 1).

Table 2 shows the birth outcomes of ART and non-ART twins. More than 60% of ART twins were born by no labour caesarean section, significantly higher than non-ART twins (46.7%). ART twins had a significantly lower stillbirth rate than non-ART twins (1.4% vs. 2.3%, *p* <  0.01) (Table 2).

**Table 2:** Birth outcomes of ART and non-ART twins, Australia, 2007–2011

	Non-ART twins (*N* = 15,082)		ART twins (*N* = 4580)		*P*-value (chi-squared test)
	No.	%	No.	%	
Delivery method					
Vaginal	4587	30.4	758	16.6	< 0.01
Labour caesarean section	3104	20.6	1011	22.1	
No labour caesarean section	7038	46.7	2764	60.3	
Not stated	353	2.3	47	1.0	
Birth status					
Live birth	14,734	97.7	4516	98.6	< 0.01
Stillbirth (fetal death)	348	2.3	64	1.4	
Preterm birth					
Yes	8585	56.9	2818	61.5	
- *Spontaneous*	*4050*	*26.8*	*1104*	*24.1*	< 0.01
- *Induced or no labor CS*	*4535*	*30.1*	*1714*	*37.4*	
No	6497	43.1	1762	38.5	

Table 3 shows the monozygotic and dizygotic rate among ART and non-ART twins. Of twins where sex is available for both babies, same-sex twins made up 55.2% of ART twins compared to 71.4% non-ART twins. According to Weinberg’s differential rule, there were 478 monozygotic ART twins and 6456 monozygotic non-ART twins. This represents a monozygotic twin rate of 2.0% for ART births and 1.1% for non-ART births (Table 3).

**Table 3:** Monozygotic and dizygotic ART and non-ART twins, Australia, 2007–2011

	Non-ART		ART	
	No.	%	No.	%
Opposite-sex twins	4300	28.6	2048	44.8
Same-sex twins	10,756	71.4	2526	55.2
Weinberg’s differential rule				
Dizygotic twins	8600		4096	
Monozygotic twins	6456		478	
All births where sex is available	582,475		24,071	
Twins where sex is available	15,056		4574	
*Dizygotic twin rate*		1.5		17.0
*Monozygotic rate*		1.1		2.0

Table 4 shows the neonatal outcomes of liveborn ART and non-ART twins. Liveborn ART twins had increased odds of low Apgar score at 5 min (< 7), preterm birth, low birthweight, requiring resuscitation, and neonatal death than liveborn non-ART twins. Any adverse neonatal outcome was present for 89.0% of liveborn non-ART twins compared to 95.3% of liveborn ART twins. After adjusted for maternal characteristics, liveborn ART twins had 28% increased odds of having any adverse neonatal outcome (AOR 1.28 95% CI 1.09–1.50) compared to non-ART liveborn twins. The sensitivity analysis by excluding BMI and smoking during pregnancy shows liveborn ART twins had 25% increased odds of having any adverse neonatal outcome (AOR 1.25 95% CI 1.07–1.47) (Table 4).

**Table 4:** Neonatal outcomes of liveborn ART and non-ART twins, Australia, 2007–2011

	Total	No.	%	OR (95% CI)	AOR^a^ (95% CI)	AOR^b^ (95% CI)
Apgar score at 5 min < 7						
Non-ART	14,718	556	3.8	1.00		
ART	4506	169	3.8	0.99 (0.83–1.18)	1.24 (1.02–1.52)	1.22 (1.01–1.50)
Preterm						
Non-ART	14,734	8281	56.2	1.00	1.00	
ART	4516	2764	61.2	1.23 (1.15–1.32)	1.13 (1.05–1.22)	1.13 (1.05–1.22)
Low birth weight						
Non-ART	14,728	7415	50.3	1.00	1.00	
ART	4515	2474	54.8	1.20 (1.12–1.28)	1.13 (1.05–1.22)	1.11 (1.03–1.20)
Resuscitation						
Non-ART	14,727	7038	47.8	1.00	1.00	
ART	4514	2569	56.9	1.44 (1.35–1.54)	1.26 (1.17–1.36)	1.29 (1.20–1.39)
Admission to intensive or special care nursery						
Non-ART	14,355	8626	60.1	1.00	1.00	
ART	4445	2945	66.3	1.30 (1.22–1.40)	1.08 (1.00–1.17)	1.09 (1.01–1.18)
Length of infant stay ≥5 days in hospital						
Non-ART	14,733	10,084	68.4	1.00	1.00	
ART	4516	3683	81.6	2.04 (1.88–2.21)	1.06 (0.97–1.17)	1.04 (0.95–1.14)
Neonatal death						
Non-ART	14,734	262	1.8	1.00	1.00	
ART	4516	93	2.1	1.16 (0.92–1.48)	1.32 (1.01–1.73)	1.31 (1.01–1.72)
Any adverse outcome						
Non-ART	14,734	13,106	89.0	1.00	1.00	
ART	4516	4304	95.3	2.52 (2.18–2.92)	1.28 (1.09–1.50)	1.25 (1.07–1.47)

^a^adjusted for maternal age, parity, Indigenous status of mother, pre-pregnancy BMI, and smoking during pregnancy

^b^adjusted for maternal age, parity, and Indigenous status of mother

Of liveborn twins where sex is available for both babies, same-sex ART twins had higher odds of preterm birth (AOR 1.19; 95% CI 1.08–1.31) and requiring resuscitation (AOR 1.28; 95% CI 1.16–1.41). The rate of any adverse neonatal outcome was slightly higher in same-sex ART twins than non-ART twins, but not statistically significant (AOR 1.20 95% CI 0.97–1.49).

For opposite-sex liveborn twins, ART twins had higher odds of Apgar score at 5 min < 7 (AOR 1.53 95% CI 1.08–2.17), preterm birth (AOR 1.37 95% CI 1.20–1.55), low birthweight (AOR 1.34 95% CI 1.18–1.52), requiring resuscitation (AOR 1.25 95% CI 1.11–1.42), and NICU/SCN admission (AOR 1.28 95% CI 1.13–1.46). Overall, opposite-sex liveborn ART twins had a 42% increased risk of having any adverse neonatal outcome compared to non-ART twins (AOR 1.42 95% CI 1.11–1.82).

## Discussion

The current analysis showed that ART twins had significantly higher rates of adverse neonatal outcomes in terms of preterm birth, low birthweight, the need for resuscitation and admission to NICU/SCN, long hospital stay and birth by caesarean section. In addition, among liveborn twins, ART twins had 28% increased odds of having any adverse neonatal outcome (AOR 1.28, 95% CI 1.09–1.50), especially for opposite-sex ART twins (AOR 1.42, 95% CI 1.11–1.82).

We found that ART mothers had higher rates of antepartum haemorrhage, pregnancy-induced hypertension, and gestational diabetes compared with non-ART mothers. This is consistent with results from two other studies [4, 9]. These adverse outcomes are partially explained by the background of subfertility or infertility. Women experiencing infertility, whether or not they undertake ART, have higher rates of many adverse outcomes when compared to women conceiving spontaneously within 12 months of trying [13].

In agreement with other studies, we found that the rate of preterm birth is significantly higher among ART twins than non-ART twins [9]. There are a number of potential explanations for the increased risk of preterm birth in ART births including the underlying maternal characteristics such as subfertility or medical conditions causing subfertility, obesity, and short stature, and the ART treatment itself [14]. The increased risk of preterm birth among twins is also partially explained by early fetal loss in a higher order multiple pregnancy [15]. Pinborg and colleagues suggested that survivors of a vanishing fetal hearts/gestational sacs were more likely to be born preterm and with low birth weight [15].

Our results illustrated that the rate of low birthweight in ART twins is significantly higher than non-ART twins. This is consistent with findings in a previous systematic review which reported a 14% increase in the risk of low birthweight among ART twins compared to non-ART twins (RR 1.14, 95% CI 1.06–1.22) [16]. The increased risk of low birthweight births in ART twins can partially be explained by the higher preterm birth rate among ART twins compared with non-ART twins. However, in this study, as in previous reports, the higher rate of preterm birth alone does not explain the increased risk of low birthweight [16, 17]. Multiple pregnancies observed on the initial ultrasound is closely linked to the increased risk of low birthweight [18].

During the study period, the twin delivery rates following ART decreased from 24.5% in 2007 to 18.2% in 2011. This has paralleled the increase in the proportion of single embryo transfer in Australia from 63.7% in 2007 to 73.2% in 2011 [6]. An Australian study suggested that a voluntary policy of single embryo transfer introduced in 2002 had a significant impact to the fall in the proportion of ART multiple births [19]. Other studies further advocated that continuing the policy of single embryo transfer would prevent multiple pregnancies and improve overall maternal and neonatal outcomes following ART [20, 21]. Luke 2017 concluded that transferring high quality and fewer embryos is responsible for reducing the risk of multiple births from ART treatments and ultimately reducing the perinatal adverse outcomes [22].

Our findings show that for opposite-sex twins, ART twins had 42% increase in the likelihood of any adverse neonatal outcome compared to non-ART twins. Opposite-sex twins are all dizygotic twins which arise from the fertilisation of two separate ova by two separate sperm resulting in the formation of two separate zygotes in natural pregnancy, or from multiple embryo transfers in ART pregnancy. Virtually all dizygotic twins develop their own placenta and membranes so are dizygotic dichorionic diamniotic twins. Exceptional cases of monochorionic dizygotic twins have been reported, however, they are very rare [23]. The higher rate of any adverse outcome in ART twins is possibly related to the underlying infertility of the couples, the development of the embryo in the laboratory or the transfers of multiple embryos [24].

Interestingly, we found that the rate of any adverse neonatal outcome was higher among same-sex ART twins than same-sex non-ART twins, but it was not statistically significant. Same sex twins can be monozygotic or dizygotic twins, given that all monozygotic twins are same-sex twins and about half of dizygotic twins are of the same sex [23]. In natural pregnancy, monozygotic twins develop by splitting zygote from the fertilisation of a single ovum with a single sperm [25]. Subsequent placentation depends on the time of zygote splitting, with monozygotic dichorionic twins if splitting before day 3 and monozygotic monochorionic twins if splitting on day 3 or after. In ART pregnancy, monozygotic twins result from the splitting of the single transferred embryo. Since embryo transfers are virtually all on day 3 or after, monozygotic monochorionic twins are likely to develop [26]. However, we are unable to identify monozygotic twins from same-sex dizygotic twins and compare the neonatal outcomes between ART monozygotic twins and non-ART monozygotic twins.

Without genetic testing and details of the number of embryos transferred available, it is difficult to identify monozygotic twins. One way to estimate the rate of monozygotic twins is to use the sex of twin pairs by applying Weinberg’s differential rule [26]. Our data showed that the proportion of same-sex twins was lower among ART group (55.2%) than non-ART group (71.4%). This proportion is slightly higher than Pinborg and colleagues proportions of same-sex twins (50.8% among ART twins and 65.3% among non-ART twins) [27] but, similar to the Australian register [28]. Based on Weinberg’s differential rule, we estimated that among ART births, monozygotic twin rate was 2.0 and 1.1% among non-ART births. These estimates are higher than the monozygotic twin rates from a systematic review where the rate was 0.9% (95% CI: 0.8–0.9%) in ART twins compared to 0.4% (95% CI: 0.4–0.4) in naturally conceived twins [29].

The major strength of this study was the population based-design. The advantage of population-based studies rests on the inclusion of all patients in a given population and are therefore have less tendency to selection biases compared to the other types of observational studies [30].

Twin pregnancies following ART treatment are highly associated with multiple embryo transfers [31]. Unfortunately, the number of embryos transferred is not captured in the NPDC. We were unable to further investigate the ART twins by single or multiple embryo transfers. In addition, there were other limitations in this study. Apart from number of embryos transferred, other detailed type of ART treatment and procedures were not available in NPDC, such as fresh versus frozen embryo transfer, cleavage versus blastocyst transfers. Studies have demonstrated that the perinatal outcomes vary by fresh versus frozen embryo transfers, autologous versus donor treatment and cleavage versus blastocyst transfers [20, 21, 32]. The information on BMI and smoking during pregnancy was not stated for a large proportion of mothers (80.4% for BMI and 25.6% for smoking during pregnancy). This reflected that some jurisdictions did not have smoking status and or BMI in their routine data collection. The large proportion of mothers with missing data on BMI and smoking during pregnancy and the differential missing data between ART and non-ART mothers would have reduced the validity of the comparison and multivariate analysis.

## Conclusion

This study using population cohort approach provided a high level of evidence where a randomized controlled trial is not feasible. The findings of this study strengthen the evidence on of higher adverse neonatal outcomes among ART twins than non-ART twins. These adverse outcomes are likely related to both the type of conception and the underlying infertility of the women. Women/couples accessing ART treatment should be fully informed of the risk of adverse outcome of twin pregnancies following ART treatment.

## Abbreviations

AIHW: the Australian Institute of Health and Welfare
AOR: Adjusted odds ratio
ART: Assisted Reproductive Technology
BMI: Body mass index
CI: Confedence interval
NICU/SCN: Neonatal intensive care unit/special care nursery
NPDC: National Perinatal Data Collections
OR: Odds ratio
SD: Standard deviation
SPSS: Statistical Package for Social Sciences
